# Retention of Minocycline Susceptibility When Gram-Positive Periprosthetic Joint Infection Isolates Are Non-Susceptible to Doxycycline

**DOI:** 10.3390/idr14050069

**Published:** 2022-08-29

**Authors:** James B. Doub, Sumon Nandi, Nicole Putnam

**Affiliations:** 1 Division of Clinical Care and Research, Institute of Human Virology, University of Maryland School of Medicine, Baltimore, MD 21201, USA; 2 Department of Orthopaedic Surgery, University of Maryland School of Medicine, Baltimore, MD 21201, USA; 3 Department of Pathology, University of Maryland School of Medicine, Baltimore, MD 21201, USA

**Keywords:** periprosthetic joint infections, minocycline, suppression antibiotics, staphylococcus, multidrug resistance

## Abstract

Background: The treatment of hardware infections often utilizes chronic oral suppression antibiotics to prevent infection recurrence. However, when methicillin-resistant *Staphylococcus aureus* and other bacteria are non-susceptible to doxycycline, limited oral antibiotic options can be available that do not cause significant side effects and drug–drug interactions. Consequently, the purpose of this study was to evaluate the ability of Gram-positive clinical prosthetic joint infection isolates that were non-susceptible to doxycycline and to retain susceptibility to minocycline. Methods: Twenty-six Gram-positive prosthetic joint infection isolates that were not susceptible to doxycycline were evaluated for retained minocycline susceptibility with the use of minocycline gradient diffusion test strips. Results: All five of the coagulase-negative staphylococcal isolates and eight of the eleven methicillin-resistant *S. aureus* isolates were susceptible to minocycline, despite being doxycycline non-susceptible. None of the five *Enterococcus faecium* PJI isolates retained susceptibility to minocycline and only two of the five *E. faecalis* isolates (*n* = 5) were susceptible to minocycline. Conclusions: The findings have direct clinical implications supporting minocycline susceptibility testing for patients with PJI and other hardware-associated infections, which have isolates that are doxycycline non-susceptible to thereby provide alternative suppression antibiotic options.

## 1. Introduction

The antibiotic resistance crisis continues to add complexities to treating certain infections. One such infectious condition that causes debilitating morbidity and increased mortality is periprosthetic joint infection (PJI) [1]. While these infections only occur in one to two percent of all arthroplasties, the projected number of annual arthroplasties continues to increase, with approximately two million surgeries to be conducted in the United States of America by 2030 [1,2]. When PJI is treated with interventions that retain infected prosthetics or with one-stage revision surgery, it is recommended for patients to receive prolonged oral suppression antibiotic therapies (SAT) [3]. The use of SAT helps prevent recurrence of PJI after intravenous antibiotic therapy thwarting the need for further surgical interventions, which thereby prevents further morbidity [3,4,5].

However, the benefits of SAT have not been robustly evaluated in clinical trials, but prolonged treatment durations have proven to be more advantageous than shorter durations in retrospective studies and are part of the PJI treatment guidelines [3,4,5]. The most common causes of PJI are *Staphylococcus aureus* and coagulase negative staphylococci (CoNS), but when these bacteria are methicillin resistant, use of SAT is relegated to sulfamethoxazole-trimethoprim, doxycycline, or linezolid [3]. If methicillin-resistant staphylococci are resistant to doxycycline, treatments are further complicated by drug–drug interactions, intolerances, and/or allergies that can limit the ability to use sulfamethoxazole-trimethoprim or linezolid. Therefore, patients needing prolonged SAT can have limited options to prevent PJI recurrence while also limiting adverse drug reactions and drug–drug interactions. However, repurposing minocycline to be an alternative antibiotic, for SAT in PJI and other hardware infections, is an attractive option to thwart these potential negative adverse reactions. Herein we examined methicillin-resistant *S. aureus* (MRSA), CoNS, *Enterococcus faecalis*, and *E. faecium* PJI isolates that were non-susceptible to doxycycline to evaluate if these isolates retained susceptibility to minocycline.

## 2. Materials and Methods

*Review and Categorization of Study Population*

This study was approved by the University of Maryland internal review board (HP-00101650). Patients were included if they had PJI between 1 January 2016 and 31 December 2020 at the University of Maryland Medical Center. Identification of patients was conducted retrospectively by query of the hospital billing database for Current Procedural Terminology (CPT) codes 27486, 27134, 27488, and 27091. PJI was defined based on Musculoskeletal Infection Society (MSIS) PJI definition [6]. A review of operating room cultures was performed for each case, and bacterial causes that had MRSA, CoNS, *E. faecalis,* or *E. faecium* that were not susceptible to doxycycline were recorded. No cases were excluded in this study.

*Evaluating Doxycycline and Minocycline Susceptibility*

All *Enterococcus spp.* isolates had been tested for tetracycline and doxycycline sensitivity with VITEK2 (Biomerieux, Marcy-l’Étoile, France), whereas *Staphylococcus* spp. had antimicrobial susceptibility testing for tetracycline performed by MicroScan (Beckman Coulter, Brea, California). Tetracycline-resistant staphylococci were reflexively tested for susceptibility to doxycycline by Kirby-Bauer disk diffusion. Isolates that were non-susceptible to tetracycline and doxycycline were unfrozen, sub-cultured onto 5% sheep blood agar plates (Hardy Diagnostics), and incubated for 24 h at 37 °C. Individual colonies for each isolate were then inoculated into sterile saline glass tubes to create a 0.5 McFarland standard, by measuring the absorbance at approximately 0.08. A sterile cotton swab was then used to streak the isolates onto Mueller-Hinton agar plates (Thermo Scientific, Waltham, MA, USA). Inoculum was allowed to absorb into the agar for 15–20 min before three minocycline gradient diffusion test strips were placed onto each agar plate. Each plate was incubated at 37 °C, and minocycline minimal inhibitory concentrations (MIC) were read 20 h later. The highest MIC value of the three gradient diffusion test strips was used to determine the MIC to minocycline. Standardized CLSI breakpoints were used to define if *Staphylococcus* spp. and *Enterococcus* isolates retained minocycline susceptibility (reference M100), where an MIC less than or equal to 4 was interpreted as susceptible, an MIC of 8 was intermediate, and an MIC of 16 or greater was resistant. Intermediate and resistant isolates are referred to herein as non-susceptible. 

## 3. Results

Table 1 shows the number of PJI cases at the University of Maryland Medical Center caused by MRSA, CoNS, *E. faecalis*, or *E. faecium* from 1 January 2016 and 31 December 2020 and the percentage that were doxycycline non-susceptible. Notably, all *E. faecium* PJI isolates (*N* = 5) were associated with polymicrobial PJI and were non-susceptible to doxycycline. CoNS isolates included *Staphylococcus epidermidis* (*N* = 18), *Staphylococcus lugdunensis* (*N* = 5), and other less frequently isolated CoNS species (all with *N* = 1): *Staphylococcus cohnii*, *Staphylococcus capitis*, and *Staphylococcus pseudointermiedius.*

Table 2 displays the minocycline susceptibility for all the PJI isolates that were non-susceptible to doxycycline. All the *E. faecium* PJI isolates (*N* = 5) were non-susceptible to minocycline, while forty percent of the *E. faecalis* isolates that were non-susceptible to doxycycline were susceptible to minocycline. Interestingly, all five of the CoNS isolates (all *S. epidermidis*) and eight of the eleven MRSA isolates (72%) were susceptible to minocycline, despite being doxycycline non-susceptible. For all isolates, the triplicate MICs were within one doubling dilution.

## 4. Discussion

Minocycline is an older tetracycline that has been repurposed to treat multidrug resistant *Acinetobacter baumannii* infections and other multidrug-resistant bacteria [7]. Furthermore, minocycline has robust activity against Gram-positive bacteria and intracellular pathogens, as well as having activity against *S. aureus* biofilms [8,9]. Prolonged minocycline use is generally well tolerated with limited side effects, making this an ideal suppression antibiotic for PJI and other hardware infections when indicated. However, there is a paucity of data with respect to minocycline activity in clinical isolates that are not susceptible to doxycycline. To our knowledge this is the first study to conduct such an evaluation.

In this brief report, we show that minocycline activity against staphylococcal PJI isolates cannot be inferred from isolates that were doxycycline non-susceptible. Rather, all the CoNS isolates and the majority of MRSA (72%) isolates tested retained susceptibility to minocycline. This has important clinical implications for treating staphylococcal PJI, suggesting that minocycline can be an alternative SAT option for PJI and other hardware infections, even when these staphylococcal isolates are not susceptible to doxycycline. Interestingly, all the *E. faecium* isolates and most of the *E. faecalis* isolates (60%) tested here were minocycline non-susceptible. Different bacterial genera, including *Staphylococcus* and *Enterococcus* species, may exhibit altered susceptibility patterns within the tetracycline class of antibiotics, given the different mechanisms of tetracycline resistance. 

There are three main mechanisms of tetracycline resistance: efflux pumps, enzymatic degradation of tetracyclines, and ribosomal protection [10]. Bacteria can acquire these by the acquisition of mobile genetic elements that have tetracycline resistance genes or through mutations in chromosomal or ribosomal subunits [10]. However, each mechanism is not associated with resistance to all tetracyclines and subsequently some tetracyclines can retain antibacterial activity against an organism that harbors resistance to other tetracyclines [10]. Genetic testing of the isolates used in this study could have helped elucidate the mechanism behind the phenotypic differences in doxycycline and minocycline susceptibilities but was not conducted given lack of funding. However, future studies could further assess this to enhance knowledge. Nonetheless, the findings seen here do not suggest all *Staphylococcus spp.* PJI isolates that are doxycycline non-susceptible will exhibit susceptibility to minocycline. Rather, because of the diverse tetracycline resistance mechanisms, testing for minocycline sensitivity can provide a potential alternative to use as SAT. Additionally, this report supports the addition of minocycline in the normal antibiogram for Gram-positive cocci causing PJI, since at the present time only doxycycline is currently tested. 

There are some limitations to our study. Firstly, the significant number of Gram-positive isolates that were non-susceptible to doxycycline were higher than expected. However, PJI patients that are treated at tertiary care centers often have recalcitrant PJI and have been on numerous treatment regimens, therefore, increasing the likelihood to develop tetracycline resistance. Secondly, while the sample size in this study was small (*n* = 26), the findings mitigate the need for larger studies given that additional minocycline susceptibility testing could be easily conducted as needed for individual cases. Lastly, this study focused on Gram-positive bacteria as these are the most common causes of PJI (3). Gram-negative bacteria were not evaluated because they are only associated with a small percent of PJI but may be worth exploring the utility of minocycline testing and treatment for other infections, such as intraabdominal infections.

## 5. Conclusions

In conclusion, minocycline is an attractive agent to use for SAT given its broad spectrum of activity and its antibacterial activity against isolates that are non-susceptible to doxycycline. The findings discussed here have direct clinical implications supporting minocycline susceptibility testing for patients with PJI and other hardware-associated infections, which have isolates that are doxycycline non-susceptible, to thereby provide alternative SAT options. As the antibiotic resistance crisis continues to worsen, repurposing older drugs and evaluating specific resistance mechanisms will be important to thwart morbidity and mortality across all infectious conditions.

## Figures and Tables

**Table 1:** PJI clinical isolates that were doxycycline non-susceptible for the four different Gram-positive bacterial groups evaluated.

Organism (*n*)	Doxycycline Susceptible Isolates *n* (%)	Doxycycline Non-Susceptible Isolates *n* (%)
MRSA (34)	18 (67.6%)	11 (32.4%)
CoNS (26)	21 (80.8%)	5 (19.2%)
*E. faecalis* (8)	3 (37.5%)	5 (62.5%)
*E. faecium* (5)	0 (0%)	5 (100%)

**Table 2:** Results of minocycline gradient diffusion testing for the PJI clinical isolates that were doxycycline non-susceptible.

Organism (*n*)	Minocycline Susceptible Isolates *n* (%)	Minocycline Non-Susceptible Isolates *n* (%)
MRSA (11)	8 (72.7%)	3 (27.3%)
CoNS (5)	5 (100%)	0 (0%)
*E. faecalis* (5)	2 (40%)	3 (60%)
*E. faecium* (5)	0 (0%)	5 (100%)

## Data Availability

The data presented in this study are available on request from the corresponding author.

## References

[1] Natsuhara K.M., Shelton T.J., Meehan J.P., Lum Z.C. (2019). Mortality During Total Hip Periprosthetic Joint Infection. J. Arthroplast..

[2] Premkumar A., Kolin D.A., Farley K.X., Wilson J.M., McLawhorn A.S., Cross M.B., Sculco P.K. (2021). Projected Economic Burden of Periprosthetic Joint Infection of the Hip and Knee in the United States. J. Arthroplast..

[3] Osmon D.R., Berbari E.F., Berendt A.R., Lew D., Zimmerli W., Steckelberg J.M., Rao N., Hanssen A., Wilson W.R. (2013). Infectious Diseases Society of America. Diagnosis and management of prosthetic joint infection: Clinical practice guidelines by the Infectious Diseases Society of America. Clin. Infect. Dis..

[4] Malahias M.-A., Gu A., Harris E.C., Adriani M., Miller A.O., Westrich G.H., Sculco P.K. (2020). The Role of Long-Term Antibiotic Suppression in the Management of Peri-Prosthetic Joint Infections Treated With Debridement, Antibiotics, and Implant Retention: A Systematic Review. J. Arthroplast..

[5] Shah N.B., Hersh B.L., Kreger A., Sayeed A., Bullock A.G., Rothenberger S.D., Klatt B., Hamlin B., Urish K.L. (2020). Benefits and Adverse Events Associated with Extended Antibiotic Use in Total Knee Arthroplasty Periprosthetic Joint Infection. Clin. Infect. Dis..

[6] Parvizi J., Tan T.L., Goswami K., Higuera C., Della Valle C., Chen A.F., Shohat N. (2018). The 2018 Definition of Periprosthetic Hip and Knee Infection: An Evidence-Based and Validated Criteria. J. Arthroplast..

[7] Ritchie D.J., Garavaglia-Wilson A. (2014). A Review of Intravenous Minocycline for Treatment of Multidrug-Resistant Acinetobacter Infections. Clin. Infect. Dis..

[8] Singh S., Khanna D., Kalra S. (2021). Minocycline and Doxycycline: More Than Antibiotics. Curr. Mol. Pharmacol..

[9] Raad I., Hanna H., Jiang Y., Dvorak T., Reitzel R., Chaiban G., Sherertz R., Hachem R. (2007). Comparative activities of daptomycin, linezolid, and tigecycline against catheter-related methicillin-resistant Staphylococcus bacteremic isolates embedded in biofilm. Antimicrob. Agents Chemother..

[10] Grossman T.H. (2016). Tetracycline Antibiotics and Resistance. Cold Spring Harb. Perspect. Med..

